# Models for successful interactions between psychiatrists and indigenous people

**DOI:** 10.1192/j.eurpsy.2021.849

**Published:** 2021-08-13

**Authors:** B. Mainguy, L. Mehl-Madrona

**Affiliations:** 1 Education Division, Coyote Institute - Canada, Ottawa, Canada; 2 Medical Arts And Humanities Program, University of Maine, Orono, United States of America

**Keywords:** Indigenous people, communication, relational self, collectivism

## Abstract

**Introduction:**

Conventional psychiatric services are not always acceptable to indigenous communities and people.

**Objectives:**

We used qualitative methodology to explore a successful collaboration of psychiatrists and addiction medicine specialists with indigenous communities in Maine, USA, in North America, comparing these results to previously unsuccessful collaborations. We wanted to delineate what leads to success.

**Methods:**

We used constant comparative, iterative methodology within a constructivist, grounded theory approach to generate differences to discuss.

**Results:**

Successful strategies address the highly relational approach to defining the self of the indigenous communities, a collectivist mindset in which the needs of the group can supersede the needs of the individual, a reliance upon stories for transmission of knowledge and culture, and a commitment to a biopsychosocial and spiritual approach, which, in North America, is often symbolized by the metaphor of the Four Cardinal Directions. Successful psychiatrists working in these communities needed to share more personal details than what they are usually accustomed to provide. They acknowledged local culture and spirituality and worked with traditional knowledge holders to create collaborative approaches. As part of this, the use of a narrative approach worked best in which the psychiatrist worked within the stories and beliefs of the community which required taking the time in dialogue to learn those stories and beliefs.

**Conclusions:**

We addressed the challenges of consulting to tribal-based treatment programs, of modifying usual counseling techniques such as motivational interviewing to an indigenous population. We propose that these sorts of participatory-action-based approaches go far to improve service delivery to indigenous people and reduce health disparities.

